# The Kinome of Edible and Medicinal Fungus *Wolfiporia cocos*

**DOI:** 10.3389/fmicb.2016.01495

**Published:** 2016-09-21

**Authors:** Wei Wei, Shaohua Shu, Wenjun Zhu, Ying Xiong, Fang Peng

**Affiliations:** ^1^Institute for Interdisciplinary Research, Jianghan UniversityWuhan, China; ^2^College of Plant Science and Technology, Huazhong Agricultural UniversityWuhan, China; ^3^College of Biology and Pharmaceutical Engineering, Wuhan Polytechnic UniversityWuhan, China; ^4^Hefei Inzyme Information Technology Co., LtdWuhan, China

**Keywords:** *Wolfiporia cocos*, edible and medicinal fungus, sclerotial development, protein kinase (PKs), kinome

## Abstract

*Wolfiporia cocos* is an edible and medicinal fungus that grows in association with pine trees, and its dried sclerotium, known as Fuling in China, has been used as a traditional medicine in East Asian countries for centuries. Nearly 10% of the traditional Chinese medicinal preparations contain *W. cocos*. Currently, the commercial production of Fuling is limited because of the lack of pine-based substrate and paucity of knowledge about the sclerotial development of the fungus. Since protein kinase (PKs) play significant roles in the regulation of growth, development, reproduction, and environmental responses in filamentous fungi, the kinome of *W. cocos* was analyzed by identifying the PKs genes, studying transcript profiles and assigning PKs to orthologous groups. Of the 10 putative PKs, 11 encode atypical PKs, and 13, 10, 2, 22, and 11 could encoded PKs from the AGC, CAMK, CK, CMGC, STE, and TLK Groups, respectively. The level of transcripts from PK genes associated with sclerotia formation in the mycelium and sclerotium stages were analyzed by qRT-PCR. Based on the functions of the orthologs in *Sclerotinia sclerotiorum* (a sclerotia-formation fungus) and *Saccharomyces cerevisiae*, the potential roles of these *W. cocos* PKs were assigned. To the best of our knowledge, our study is the first identification and functional discussion of the kinome in the edible and medicinal fungus *W. cocos*. Our study systematically suggests potential roles of *W. cocos* PKs and provide comprehensive and novel insights into *W. cocos* sclerotial development and other economically important traits. Additionally, based on our result, genetic engineering can be employed for over expression or interference of some significant PKs genes to promote sclerotial growth and the accumulation of active compounds.

## Introduction

*Wolfiporia cocos* (Schwein.) Ryvarden *et* Gilb. (Basidiomycota, Polyporaceae) is an edible fungus, and its sclerotia, known as Fuling in China, have been reported to possess important medicinal value (Dai et al., 2009; Esteban, 2009; Lin et al., 2009; Kobira et al., 2012; Wang et al., 2013). Pharmacological research pertaining to the two major active compounds from *W. cocos* sclerotia, polysaccharides and triterpenes, has demonstrated their multiple immune stimulatory and pharmacological activities (Rios, 2011; Feng et al., 2013; Wang et al., 2013, 2015a,b; Zhao et al., 2013; Tang et al., 2014; Gao et al., 2016).

Sclerotial formation of *W. cocos* is dependent on colonization of *Pinus* species (Wang et al., 2002; Kubo et al., 2006; Xiong et al., 2006; Xu et al., 2014). Therefore, commercial production of *W. cocos* sclerotia consumes a significant amounts of Pinus wood each year (Wang et al., 2012). In order to support efforts to improve the yield and efficiency of *W. cocos* sclerotia, the present study aimed to further knowledge of the regulatory mechanisms operating in the fungus.

In eukaryotic organisms, especially fungi, protein kinases (PKs) catalyze reversible phosphorylation of serine, threonine or tyrosine residues to control the activity of functional proteins, and they play significant roles in regulating growth, reproduction, developmental processes, and environmental stress responses (Cohen, 2000; Turrà et al., 2014). For example, in *Sclerotinia sclerotiorum*, a plant pathogenic fungus, silencing the mitogen-activated PK (MAPK) gene *SMK1* resulted in impaired sclerotial formation (Chen et al., 2004). Sclerotia development of *S. sclerotiorum* is also associated with increased cAMP-dependent PK A (PKA) levels (Rollins and Dickman, 1998; Harel et al., 2005; Jurick and Rollins, 2007), indicating that the cAMP-PKA signaling pathway is important in the regulation of sclerotial development. In *Botrytis cinerea*, another plant pathogenic fungus, the HOG1-type MAPK BcSAK1 is involved not only in the response to osmotic stress but also in sclerotial development (Segmüller et al., 2007) and deletion of its *bmp3* gene encoding a homolog of the yeast MAPK Slt2 results in the loss of sclerotial formation (Rui and Hahn, 2007).

In the present study, 103 putative PK genes were putatively identified in the *W. cocos* genome based on homologous sequences searching by using BLASTx program against the *Saccharomyces cerevisiae* and *S. sclerotiorum* databases. Based on known and presumed functions of the orthologs of these PK genes found in other fungi, the putative roles of these *W. cocos* PKs in colonization, mycelial growth, development and response to environmental stress were assigned. The data from our study contribute to a better understanding of the potential roles of PKs in various processes of *W. cocos* and will help to illuminate the mechanisms of sclerotial formation.

## Materials and Methods

### Strains and Culture Conditions

**FIGURE 1:**
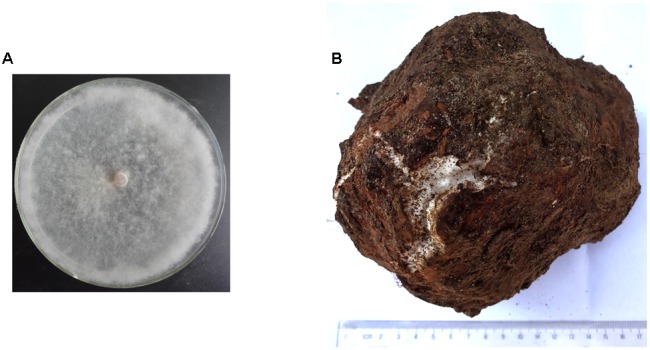
**The mycelium and sclerotia of *Wolfiporia cocos* used in this study. (A)** Colony morphology *W. cocos* mycelium grown on PDA for 7 days at 25°C. **(B)** *W. cocos* mature sclerotium (6-months-old).

Sclerotium and mycelium of *W. cocos* (**Figure 1**) collected from Yingshan county, Hubei province, China (Shu et al., 2013) were kindly donated by Dr. Shaohua Shu at the Huazhong Agricultural University. *W. cocos* mycelium was grown on a cellophane membrane place on the surface of potato dextrose agar (PDA) medium at 28°C for 7 days. Both the mycelium and sclerotium were frozen in liquid nitrogen and stored at -80°C for total RNA extraction.

### Identification of PKs

RNA-seq data from two samples of mycelium and sclerotium was from a previous study (Shu et al., 2013) and is accessible under accession number SRP018935 at NCBI^1^. Genome data was retrieved from the JGI database^2^.

Homologous sequence searches were performed with BLASTx against the *S. cerevisiae* database ^3^ and *S. sclerotiorum* database^4^ (≥e^-5^) as described (Hegedus et al., 2016; Zhang et al., 2016).

To identify and classify the PKs, the protein sequences from *W. cocos* were searched against the Kinomer v.1.0 HMM library by the using of HMMSCAN program from the HMM software suite HMMer, and the cut off value was set to 20 as previously described (Miranda-Saavedra and Barton, 2007; Kosti et al., 2010; Wang et al., 2011; Zhang et al., 2016).

### Differential Expression Analysis

The RPKM method (Reads per kb per Million reads) was used to calculate RNA levels as previously described (Zhang et al., 2016; Mortazavi et al., 2008; Shu et al., 2013). To identify differentially expressed genes (DEGs) in mycelium and sclerotium, the statistical method of false discovery rate (FDR) was employed to correct the threshold of *P*-value in multiple tests. Those DEGs with a ratio ≥ 2 and an FDR ≤ 0.001 were chosen for this study. As previously described, the DEGs were analyzed with DEGSeq (Wang et al., 2010).

### RNA Extraction and qRT-PCR Confirmation of PKs Gene Transcription

Total RNA of mycelium and sclerotium were extracted with TriZOL reagent (Invitrogen^TM^, Carlsbad, CA, USA) and treated with RNase-free DNase I (Qiagen Inc, Duesseldorf, Germany) to remove residual DNA according to manufacturer’s protocols. The High Capacity cDNA Reverse Transcription Kit (Applied Biosystems^TM^, Foster, CA, USA) was used to generate the first strand cDNA according to the manufacturer’s instructions. Gene expression was analyzed by qRT-PCR using a Bio-Rad CFX96 Real Time System (Bio-Rad, America) and SYBR Premix Ex Taq II (TAKARA, Dalian, China), according to the manufacturer’s instructions. The PCR conditions were as follows: denaturation at 95°C for 3 min; 40 cycles of 95°C for 15 s, 55°C for 15 s and 72°C for 30 s; final step of 72°C for 10 min. The primers for qRT-PCR are listed in **Table 1**. The mRNA levels of the *W. cocos* alpha-tubulin gene (Shu et al., 2013; Zhang et al., 2016) were used to normalize the data for each qRT-PCR run. For each gene, qRT-PCR assays were repeated at least three times, with each repetition having three technical replicas.

**Table 1:** Primers used for qRT-PCR.

Primers used for qRT-PCR	
TPK2 (Wolco1| 107737)	TPK2 F: 5′ TCGTCAAAATGAAGCAGGTCT 3′
	TPK2 R: 5′ CGAAGAAGAGTGAATAGCTCCC 3′
HOG1(Wolco1| 95503)	HOG1 F: 5′ CAAACCGAGCAATATCCTAATC 3′
	HOG1 R: 5′ TGATCTCGGGCGCGCGGTAG 3′
FUS3 (Wolco1| 107181)	FUS3 F: 5′ ATGCTCAATACTTCATCTACCAAAC 3′
	FUS3 R: 5′ GACCGAGCGTGCGAGACCG 3′
SLT2 (Wolco1| 92114)	SLT2 F: 5′ GTGTGGTCCGTCGGATGCATT 3′
	SLT2 R: 5′ CGATGGCGTACCGAGGTAGT 3′
CLA4 (Wolco1| 121529)	CLA4 F: 5′ CGCCCGAGTCGTTGCGTC 3′
	CLA4 R: 5′ CATGATGGTCGCAGGCATCAC 3′
STE20 (Wolco1| 166770)	STE20 F: 5′ CGTACCAGCTCGGGACGAAC 3′
	STE20 R: 5′ CGAGTCGATGTAGTTTACGATGTTG 3′
STE11(Wolco1| 43954)	STE11 F: 5′ GGATGGACGCTTCAACGGGCTT 3′
	STE11 R: 5′ TCGTCAATGCATGAAGAGAGATACT 3′
STE7 (Wolco1| 150169)	STE7 F: 5′ ACGTCCCGACGGGCACGAT 3′
	STE7 R: 5′ TTGTCCATGAACTCCATGCAGATAC 3′
MKK2 (Wolco1| 75024)	MKK2 F: 5′ GCCGTCCAGCAGCGAGGT 3′
	MKK2 R: 5′ GAATGGTCTTCATCTTGTGGAGGT 3′
Alpha-tubulin	Alpha-tubulin F: 5′ ACTCCAGCTTGGACTTCTTG 3′
	Alpha-tubulin R: 5′ TCTTCGTCTTCCACTCCTTTG 3′

## Results

### Predicted PKs in *W. cocos*

Our search of the *W. cocos* genome sequence identified a total of 103 putative PKs, 87 of them with significant similarity (≥e^-5^) to one or more of the 92 *S. sclerotiorum* and/or 115 *S. cerevisiae* PKs (**Supplementary Table S1**) (Hunter and Plowman, 1997; Hegedus et al., 2016). On the basis of conserved residues, 11 candidates belonged to the atypical PKs, 13 to the AGC Group, 10 to the CAMK Group, 2 candidates to the CK Group, 22 candidates to the CMGC Group, 11 to the STE Group, 10 to the TLK Group, and 24 to the Other Group (**Supplementary Table S1**). Compared with *S. cerevisiae, W. cocos* is predicted to have fewer PKs genes. It lacks orthologs of 30 *S. sclerotiorum* and/or *S. cerevisiae* PKs, including DBF2/4, ELM1, ALK1/2, PTK1/2, NPR1, HSL1, ISR1, YGR052W, YLR253W, YMR291W, HAL5, KKQ8, NNK1, PRR1/2, YPL150W, KCC4, MEK1, DUN1, KSP1, CAK1, SRB10, SSK2/SSK22, MPS1, PSK1/2, BUD32, TOS3, SAK1, SCY1, IKS1/2, and RAD53 (**Supplementary Table S1**). Moreover, *W. cocos* has two orthologs of CBK1 (Wolco1| 74908 and Wolco1| 70675), COQ8 (Wolco1| 138133 and Wolco1| 144299), PKP1 (Wolco1| 110818 and Wolco1| 92641), TEL1 (Wolco1| 108405 and Wolco1| 91919), VHS1 (Wolco1| 106038 and Wolco1| 155089), CDC28 (Wolco1| 91184 and Wolco1| 93694), SAT4 (Wolco1| 159823 and Wolco1| 134885), EVN7 (Wolco1| 137941 and Wolco1| 132412), ATG1 (Wolco1| 20796 and Wolco1| 109315), SPS1 (Wolco1| 95124 and Wolco1| 111584) and three orthologs of YAK1 (Wolco1| 64270, Wolco1| 97250 and Wolco1| 75698), SKY1 (Wolco1| 159141, Wolco1| 92467 and Wolco1| 95712), Pan3p (Wolco1| 164201, Wolco1| 138179 and Wolco1| 73826) (**Supplementary Table S1**). *W. cocos* also contains 16 putative PKs that have no distinct orthologs in *S. cerevisiae* and/or *S. sclerotiorum*.

### AGC Group

The *W. cocos* AGC Group has 13 members. The PKs from the AGC Group are important in the regulation of signaling pathways in response to cell wall or membrane stress and limited nutrients. In *S. cerevisiae*, in response to limited nutrients and other stresses, the activity of YPK1/2, SCH9, RIM15, DBF2/20, TPK1/2 (PKA catalytic subunit) and PKC1 are directly or indirectly regulated by the atypical PKs TOR1 and TOR2 (the target of rapamycin; Jacinto and Lorberg, 2008; Turrà et al., 2014).

Like many other filamentous fungi, *W. cocos* contains two genes, Wolco1| 115209 and Wolco1| 107737, that encode the catalytic subunits of PKA, similar to *S. cerevisiae* TPK1 and TPK2, and *S. sclerotiorum* SS1G_03171 and SS1G_13577, respectively. In *Fusarium graminearum*, deletion of the PKA-encoding gene *cpk1* causes significantly decreased vegetative growth, conidiation and deoxynivalenol production. Furthermore, the *cpk1* mutant was also defective in ascospore maturation and releasing. In contrast, the mutant of another PKA-encoding gene, *cpk2*, had no detectable phenotypes (Hu et al., 2014). In *S. cerevisiae*, given elevated glucose levels, the activated PKA regulates SNF1 (Wolco1| 160811) activity, thereby regulating downstream signaling pathways (Barrett et al., 2012). In *S. sclerotiorum*, deletion of SS1G_03171 did not result in an altered phenotype (Jurick et al., 2004), indicating the functional redundancy of the PKA. In the present study, mRNA levels originating from *W. cocos* PKA Wolco1| 107737, which is homologous to *S. cerevisiae* TPK2 (NP_015121.1) and *S. sclerotiorum* SS1G_13577, increased at the sclerotia stage. However, the mRNA level of the other PKA, Wolco1| 115209, which is homologous to *S. cerevisiae* TPK1 (NP_012371.2) and *S. sclerotiorum* SS1G_03171, did not change (**Supplementary Table S1**), indicating that Wolco1| 107737, but not Wolco1| 115209, may play a major role in the sclerotial development of *W. cocos*. The aurora kinase *IPL1* (NP_015115.1) of *S. cerevisiae* and Fg06959 of *F. graminearum* are essential; their deletion is lethal (Wang et al., 2011). However, in *W. cocos*, there was no difference in mRNA levels for this kinase between the mycelium and sclerotia stages (**Supplementary Table S1**), indicating that *W. cocos* IPL1 may not be involved in sclerotial formation.

*Wolfiporia cocos* Wolco1| 71589 and Wolco1| 24527 encode PKs orthologous to *S. cerevisiae* SCH9 and RIM15, respectively. SCH9 is structurally related to PKA and works with PKA to negatively control RIM15 activity, thereby regulating the response to nutrient starvation or stress in yeast (Roosen et al., 2005). In *F. graminearum*, Fgsch9 (Fg00472) is involved in both deoxynivalenol (DON) production and growth, and Fgrim15 (Fg01312) plays important roles in DON production and conidiation (Wang et al., 2011). In the current study, *W. cocos SCH9* (Wolco1| 71589) demonstrated higher mRNA levels in the sclerotial than the mycelial stage. In contrast, mRNA levels for *W. cocos RIM15* (Wolco1| 24527) decreased at the sclerotial stage (**Supplementary Table S1**).

Several AGC Group PKs, including YPK1/YPK2, PKC1 and SCH9, are phosphorylated and activated by PHK1/PHK2 (Roelants et al., 2004). In *S. cerevisiae*, YPK1/YPK2 are involved in cell-wall integrity (Roelants et al., 2002), and YPK1 also phosphorylates and down regulates Fpk1 kinase activity (Roelants et al., 2010). In *F. graminearum*, deletion of *Fgfpk1* (*Fg04382*) results in a reduced growth rate and increased sensitivity to osmotic and oxidative stress (Wang et al., 2011). PKC1 (PKC) targets BCK1 and regulates cell-wall integrity SLT2 MAPK (Reinoso-Martín et al., 2003; Turrà et al., 2014), and PKC1 is an essential gene in both *F. graminearum* and yeast. CBK1 is essential in wild-type *S. cerevisiae* strains and is involved in the regulation of polarized growth and cell-wall integrity by regulating the activity of SDP1 (Kurischko et al., 2005; Kuravi et al., 2011). In *F. graminearum*, the growth rate of *CBK1* (Fg01188) mutants decreased by more than 90% and mutant strain formed compact colonies (Wang et al., 2011) (**Table 2**). In *W. cocos*, there are two PKs; one of them (Wolco1| 70675) is highly similar to the yeast CBK1 (NP_014238.3, *E*-value: 0.00E), indicating their potential roles in growth and cell-wall integrity (**Table 2**).

**Table 2:** Potential functions of AGC Group PKs.

Protein ID	Orthologs	Potential functions
Wolco1| 115209	TPK1	Growth, conidiation, deoxynivalenol (DON) production, ascospore maturation and releasing
Wolco1| 107737	TPK2	Sclerotial development
Wolco1| 88682	IPL1	Essential gene
Wolco1| 71589	SCH9	Response to nutrient starvation or stress, growth and DON production
Wolco1| 24527	RIM15	DON production and conidiation
Wolco1| 29283	YPK1	Cell-wall integrity, growth and response to osmotic and oxidative stresses
Wolco1| 97232	YPK2	Cell-wall integrity
Wolco1| 16337	PKC1	Cell-wall integrity and essential gene
Wolco1| 74908	CBK1	Growth, cell-wall integrity and essential gene
Wolco1| 70675	CBK1	Growth, cell-wall integrity and essential gene

### CAMK Group

The *W. cocos* CAMK Group has 10 members, eight of which are similar to *S. cerevisiae* CAMK-like (CAMKL) kinases (**Supplementary Table S1**; **Table 3**). *Wolco1| 160811* encodes sucrose non-fermenting (SNF1) kinase, and its homolog in *S. sclerotiorum* and *S. cerevisiae* are involved in carbon catabolite repression (Sanz, 2003; Vacher et al., 2003). In the plant pathogenic fungus *Gibberella zeae*, GzSNF1 is important for vegetative growth, sexual reproduction and pathogenesis (Lee et al., 2009). Wolco1| 19611 is most similar to *S. cerevisiae* KIN1, which regulates exocytosis (Elbert et al., 2005). The homolog of Wolco1| 153019 in *F. graminearum* Fgkin4 is involved in growth, septum formation, conidiation and sexual reproduction (Wang et al., 2011), and its homolog in *S. cerevisiae* KIN4 plays a key role in the regulation of the spindle position checkpoint of cells exiting mitosis (Caydasi et al., 2010). Kinase Chk1 functions in DNA damage checkpoint in eukaryotes (Sanchez et al., 1999). In *F. graminearum*, FgChk1 (Fg01506) is important for DNA damage repair but not necessary for pathogenesis deletion (Wang et al., 2011). *Wolco1| 137455* encodes kinase similar to *S. sclerotiorum* SS1G_06203, and *S. cerevisiae* RCK2. RCK2 is targeted by HOG1 and links to the pheromone response and hyperosmotic stress via MAPK pathways (Nagiec and Dohlman, 2012).

**Table 3:** Potential functions of CAMK Group PKs.

Protein ID	Orthologs	Potential functions
Wolco1| 160811	SNF1	Carbon catabolite repression, growth, sexual reproduction and pathogenesis
Wolco1| 19611	KIN1	Exocytosis
Wolco1| 153019	KIN4	Cells mitosis
Wolco1| 92282	CHK1	DNA damage repair
Wolco1| 137455	RCK2	Pheromone response and hyperosmotic stress

### CMGC Group

The *W. cocos* CMGC Group has 22 members, including the homolog of MAPKs (**Supplementary Table S1**; **Table 4**). MAPK cascades and MAPK signaling pathways are known to be involved in many major cell processes in fungi (Turrà et al., 2014). We found three MAPKs in *W. cocos*. Wolco1| 95503 orthologous to the HOG1-style MAPK in *S. sclerotiorum* (SS1G_07590) and *S. cerevisiae* (NP_013214.1) with high similarity. In *B. cinerea*, the closely related phytopathogen of *S. sclerotiorum*, the HOG1 ortholog is involved in osmotic stress, oxidative stress, conidia formation and sclerotial development (Segmüller et al., 2007). Wolco1| 107181 is orthologous to the FUS3-style MAPK SMK1 (SS1G_11866) in *S. sclerotiorum*. Silencing of *SMK1* in *S. sclerotiorum* results in impaired sclerotial formation (Chen et al., 2004). Wolco1| 92114 encodes a kinase similar to *S. sclerotiorum* SMK3 (SS1G_06203) and *S. cerevisiae* SLT2-style MAPK (NP_011895.1). Deletion of *SMK3* in *S. sclerotiorum* inhibits the production of sclerotia (Bashi et al., 2010). Deletion of the *B. cinerea bmp3* gene encoding a homolog of the yeast MAPK Slt2 also results in the loss of sclerotial formation (Rui and Hahn, 2007).

**Table 4:** Potential functions of CMGC Group PKs.

Protein ID	Orthologs	Potential functions
Wolco1| 95503	HOG1	Osmotic stress, oxidative stress, conidia formation and sclerotial development
Wolco1| 107181	FUS3	Sclerotial formation
Wolco1| 92114	SLT2	Sclerotial formation
Wolco1| 91184	CDC28	Cell division
Wolco1| 93694	CDC28	Cell division
Wolco1| 99718	CTK1	Growth, conidiation, sexual reproduction and infection
Wolco1| 93172	PHO85	Cell division, response to nutrient levels and environmental stresses, cell cycle control and morphogenesis
Wolco1| 64270	YAK1	Cell proliferation, differentiation, homeostasis, and response to H_2_O_2_
Wolco1| 97250	YAK1	Cell proliferation, differentiation, homeostasis, and response to H_2_O_2_
Wolco1| 75698	YAK1	Cell proliferation, differentiation, homeostasis and response to H_2_O_2_
Wolco1| 64147	IME2	Meiosis initiation and spore formation
Wolco1| 159141	SKY1	Metabolic signaling, cell-cycle regulation, and chromatin reorganization
Wolco1| 92467	SKY1	Metabolic signaling, cell-cycle regulation, and chromatin reorganization
Wolco1| 95712	SKY1	Metabolic signaling, cell-cycle regulation, and chromatin reorganization

Besides MAPKs, other members of CMGC Group kinases also play important roles in many major cell processes. Wolco1| 91184 and Wolco1| 93694 are the orthologs of yeast CDC28 (NP_009718.3) that regulate cell division in eukaryotes (Malumbres, 2014). Wolco1| 99718 is the ortholog of yeast CTK1(NP_012783.1) that is involved in sexual reproduction in yeast. In *F. graminearum*, orthologs of CTK1 Fgctk1 are involved in growth, conidiation, sexual reproduction and infection (Wang et al., 2011). *W. cocos* Wolco1| 93172 encodes PKs orthologous to *S. cerevisiae* PHO85. Deletion of *PHO85* is not lethal in *S. cerevisiae*, whereas its ortholog is essential in *Ustilago maydis* and *Cryptococcus neoformans* (Castillo-Lluva et al., 2007; Virtudazo et al., 2010). In yeast, PHO85 is involved in the regulation of cell division in response to nutrient levels and environmental stresses (Nishizawa, 2015). In *Aspergillus nidulans*, the homologs of PHO85 play an essential role in cell cycle control and morphogenesis (Dou et al., 2003).

*Wolfiporia cocos* has three orthologs of YAK1 (Wolco1| 64270, Wolco1| 97250 and Wolco1| 75698). The YAK1 members control cell proliferation, differentiation and homeostasis (Aranda et al., 2011). In *F. graminearum*, the *yak1* mutant is more sensitive to H_2_O_2_ than the wild type strain (Wang et al., 2011). Wolco1| 64147 is an ortholog of yeast IME2 (NP_012429.1), which is essential for meiosis initiation (Honigberg, 2004). It also regulates spore formation in response to nutrient levels and cAMP (McDonald et al., 2009). *W. cocos* has three orthologs of SKY1 (Wolco1| 159141, Wolco1| 92467 and Wolco1| 95712). SKY1 is a serine-arginine PK that is involved in metabolic signaling, cell-cycle regulation and chromatin reorganization (Giannakouros et al., 2011). In *F. graminearum*, the *sky1* mutant was reduced in hyphal branching and produced fewer aerial hyphae (Wang et al., 2011).

### STE Group

The *W. cocos* STE Group contains 11 members (**Supplementary Table S1**; **Table 5**). Wolco1| 166770, Wolco1| 43954 and Wolco1| 150169 are the orthologs of yeast STE20, STE11, and STE7, respectively. In the MAPK cascade, MAPKKs (STE7) phosphorylate MAPKs, MAPKKKs (STE11) phosphorylate MAPKKs (STE7) and STE20 is the upstream kinase (Gustin et al., 1998; Chen and Thorner, 2007). Deletion of *STE7* and *STE11* orthologs in *B. cinerea* results in reduced growth and virulence (Schamber et al., 2010). The BMP1 MAPK mutant of *B. cinerea* was unable to form sclerotia and exhibited decreased virulence (Doehlemann et al., 2006). Wolco1| 75024 is orthologous to *S. cerevisiae* MKK2 MAPKKs, which act in the upstream SLT2-style MAPK pathway and activate this signaling pathway. Wolco1| 144702 is orthologous to *S. cerevisiae* PBS2 MAPKKs, which act upstream of the HOG1-style MAPK pathway. Wolco1| 135363 is orthologous to *S. cerevisiae* BCK1, which acts upstream of the SLT2-style MAPK pathway (Turrà et al., 2014). Finally, the CLA4 orthologs in *M. grisea* and *Claviceps purpurea* are involved in pathogenicity, mycelial growth and conidial morphology (Li et al., 2004; Rolke and Tudzynski, 2008).

**Table 5:** Potential functions of STE Group PKs.

Protein ID	Orthologs	Potential functions
Wolco1| 166770	STE20	Growth, virulence, and sclerotial formation
Wolco1| 43954	STE11	Growth, virulence, and sclerotial formation
Wolco1| 150169	STE7	Growth, virulence, and sclerotial formation
Wolco1| 75024	MKK2	Regulate MAPK signaling pathway
Wolco1| 144702	PBS2	Regulate MAPK signaling pathway
Wolco1| 135363	BCK1	Regulate MAPK signaling pathway
Wolco1| 121529	CLA4	Pathogenicity, mycelial growth and conidial morphology

### CK Group

There are only two members of the *W. cocos* CK Group (**Supplementary Table S1**; **Table 6**). Wolco1| 22440 is orthologous to *S. cerevisiae* HRR25. HRR25 is a multifunctional kinase that is involved in autophagy and endocytosis (Tanaka et al., 2014; Peng et al., 2015). The other CK member, Wolco1| 152548, encodes a PK similar to the *S. cerevisiae* PKs YCK1/YCK2. YCK1/YCK2 are membrane-localized kinases that phosphorylate another membrane anchor protein OPY2 and active the HOG1 signaling pathway in response to high-glucose conditions (Yamamoto et al., 2010).

**Table 6:** Potential functions of CK Group PKs.

Protein ID	Orthologs	Potential functions
Wolco1| 22440	HRR25	Autophagy and endocytosis
Wolco1| 152548	YCK1/YCK2	Response to high-glucose conditions

### TKL Group

There are 10 members in the *W. cocos* TKL Group. However, their homologous PKs are not typically found in *S. sclerotiorum* and *S. cerevisiae*, indicating that the TKL Group may only exist in higher fungi and eukaryotes.

### Other Group

There are 24 PKs members in Other Group that do not cluster into the major groups described above (**Supplementary Table S1**; **Table 7**). Wolco1| 159823 and Wolco1| 134885 are orthologous to *S. cerevisiae* SAT4, which is involved in regulating permeases activity and salt tolerance (Mulet et al., 1999; Pérez-Valle et al., 2010). In *F. graminearum*, the Fgsat4 mutant was more sensitive to 0.7 M NaCl but more tolerant to 0.7 M KCl (Wang et al., 2011). HRK1 (Wolco1| 142396) contributes to regulating membrane ATPase activity, which is important for glucose uptake (Goossens et al., 2000). Wolco1| 19350 is orthologous to SKS1, which is involved in the response to glucose and hyphal development (Johnson et al., 2014). BUB1 (Wolco1| 144048) and ARK1 (Wolco1| 130299) are involved in the formation of the mitotic spindle pole, cytokinesis, chromosome orientation and separation (Leverson et al., 2002; Storchová et al., 2011). There are six members in this group; however, their homologous PKs are not typically found in *S. sclerotiorum* and *S. cerevisiae*.

**Table 7:** Potential functions of other group PKs.

Protein ID	Orthologs	Potential functions
Wolco1| 159823	SAT4	Permeases activity and salt tolerance
Wolco1| 134885	SAT4	Permeases activity and salt tolerance
Wolco1| 142396	HRK1	Regulation of membrane ATPase activity
Wolco1| 19350	SKS1	Response to glucose and hyphal development
Wolco1| 144048	BUB1	Regulate the formation of mitotic spindle pole, cytokinesis, chromosome orientation and separation
Wolco1| 130299	ARK1	Regulate the formation of mitotic spindle pole, cytokinesis, chromosome orientation and separation

### qRT-PCR of mRNA from Genes Involved in Sclerotia Formation

**FIGURE 2:**
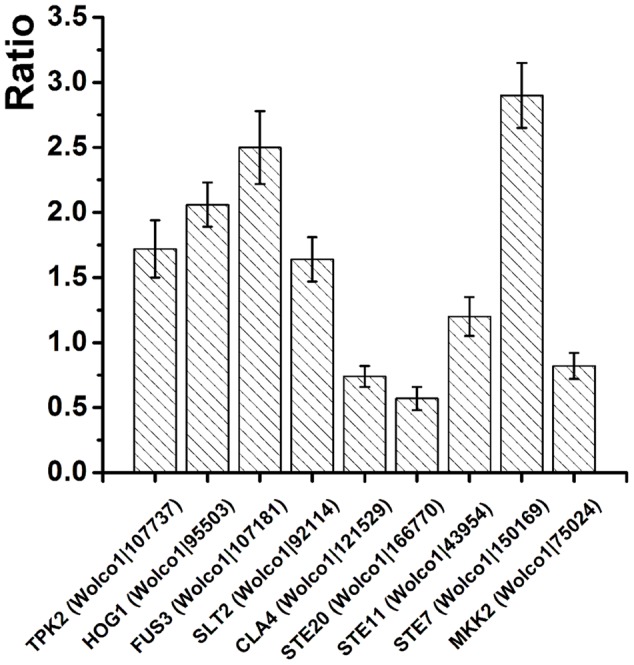
**qRT-PCR validation of sclerotia-formation associated genes.** The relative expression of target genes in mycelium stage was set as level 1. Expression levels of *W. cocos* alpha-tubulin gene was used to normalize different samples. Bars represent means and standard deviations (three replications). *Y*-axis represent the ratio of genes expression in sclerotial stage to mycelium stage.

Nine *W. cocos* PKs whose orthologs in other fungi are involved in sclerotial formation were chosen for a validation procedure of their RNA levels were determined via qRT-PCR. The results revealed that mRNAs from genes encoding TPK2 (Wolco1| 107737), HOG1 (Wolco1| 95503), FUS3 (Wolco1| 107181), SLT2 (Wolco1| 92114), STE11 (Wolco1| 43954) and STE7 (Wolco1| 150169) were present in higher amounts during the sclerotial stage; CLA4 (Wolco1| 121529), STE20 (Wolco1| 166770), and MKK2 (Wolco1| 75024) mRNAs were present in lower amounts (**Figure 2**). These results are consistent with the *de novo* transcriptome sequencing data (**Supplementary Table S1**), indicating that *W. cocos* PKs regulate sclerotial formation in both positive and negative ways.

## Discussion

Since shortages in pine wood resources currently limit the commercial production of *W. cocos* sclerotia, it is important to find ways to improve the sclerotial yield and their content of pharmacologically active components. We hypothesize that better knowledge of the kinome of the fungus can point to ways of achieving this goal.

In *S. sclerotiorum*, sclerotial formation is dependent on mycelial differentiation and the response to stress, nutrient and environmental changes via PK signaling pathways (Erental et al., 2008). Silencing of the FUS3-style MAPKs gene *SMK1* results in impaired sclerotial formation (Chen et al., 2004). In *W. cocos*, Wolco1| 107181 is orthologous to SMK1, and its expression level is upregulated at the sclerotial stage (**Figure 2**; **Supplementary Table S1**). Besides *SMK1*, Wolco1| 92114, which is orthologous to SLT2-style MAPKs, is also upregulated in the sclerotial stage (**Figure 2**; **Supplementary Table S1**), indicating that MAPKs also potentially positively regulate sclerotial formation in *W. cocos*. In *B. cinerea*, deletion of the *bmp3* gene encoding a homolog of the yeast SLT2-style MAPKs results in the loss of sclerotial formation (Rui and Hahn, 2007). Additionally, like FUS3-style and SLT2-style MAPKs, the HOG1-type MAPK BcSAK1 is also involved in sclerotial development (Segmüller et al., 2007), and the homolog of HOG1-type MAPK in *W. cocos* is also upregulated (**Figure 2**; **Supplementary Table S1**), suggesting that MAPKs play significant roles in the sclerotial formation of multiple fungi. In addition, sclerotial development of *S. sclerotiorum* is also associated with the cAMP-PKA signaling pathway in a complicated way (Rollins and Dickman, 1998; Harel et al., 2005; Jurick and Rollins, 2007). *W. cocos* contains two genes, *Wolco1| 115209* and *Wolco1| 107737*, that encode the catalytic subunits of PKA, similar to *S. sclerotiorum SS1G_03171* and *SS1G_13577*, respectively. In *S. sclerotiorum*, deletion of *SS1G_03171* does not result in an altered phenotype (Jurick et al., 2004). In the current study, the expression level of *W. cocos Wolco1| 107737* was upregulated at the sclerotia stage, and the expression level of the other PKA *Wolco1| 115209* did not change significantly (**Supplementary Table S1**), indicating that Wolco1| 107737, but not Wolco1| 115209, potentially plays the major role in sclerotial development in *W. cocos*.

Although sclerotial development has recently been studied (Wu et al., 2016; Zhang et al., 2016), the mechanisms of sclerotial development and *W. cocos*-pine wood interactions remain largely unknown. This paucity of knowledge is also the case particularly for sclerotogenesis, which is regulated via PK signaling pathways. In addition, since *W. cocos* is only able to form sclerotia after colonization of pine wood, we hypothesize that some components from pine woods may induce mycelial differentiation and sclerotial development via PK signaling pathways. Since multiple PK genes whose homologs regulate sclerotial formation in other fungi were upregulated in mature sclerotia of *W. cocos*, it is likely that sclerotogenesis and sclerotial development occur in response to stress and/or nutrient and environmental change via PK signaling pathways.

Because PK pathways integrate multiple external and internal signals to co-regulate the key processes of the fungal life cycle, such as growth, infection, nutrient or stress responses, metabolism and sclerotial development (Erental et al., 2008; Turrà et al., 2014), over-expression of key PK genes or interference in their expression will change physiological processes and traits. In *Ganoderma lucidum*, another traditional Chinese medicinal mushroom, over-expression of the 3-hydroxy-3-methylglutaryl coenzyme A reductase gene by using *Agrobacterium tumefaciens*-mediated transformation method led to a two fold increase in ganoderic acid content, increased accumulation of intermediates and the up-regulation of downstream genes (Xu et al., 2012), indicating that genetic engineering is an efficient approach to manipulate the economic traits of fungi. Furthermore, an efficient and stable genetic transformation system for *W. cocos* has been developed (Sun et al., 2015), and the genes and pathways involved in triterpenoids (the main active compound in *W. cocos*) biosynthesis are known (Shu et al., 2013). Based on our analysis, the orthologs of TPK2, HOG1, FUS3, SLT2, STE11, and STE7 in other fungi play significant roles in sclerotial development, and they are upregulated during the sclerotial stage (**Figure 2**; **Supplementary Table S1**), indicating that these PKs potentially positively regulate sclerotial formation in *W. cocos*. Therefore, we have selected these six PKs as the target genes for eventual over-expression in *W. cocos* by genetic engineering to improve the sclerotial yield.

In summary, we have identified and discussed the potential roles of *W. cocos* PK genes in growth, sclerotial developmental, morphological changes and environmental stress responses. Characterization of these PK genes will help illuminate the underlying mechanisms of sclerotogenesis and improve the sclerotial yield.

## Conclusion

This study firstly contributes to an understanding of putative functions of PKs in *W. cocos* sclerotial development and other important physiological processes. And it also provides a valuable data for illuminating the mechanisms of *W. cocos* sclerotial development and promoting commercial cultivation of *W. cocos* sclerotia.

## Author Contributions

Conceived and designed the experiments: WZ and WW. Performed the experiments: WW, SS, and WZ. Analyzed the transcription data: WW, WZ, and YX. Contributed reagents/materials/analysis tools: WW, SS, WZ, YX, and FP. Wrote the paper: WZ and WW. All authors read and approved the final manuscript.

## Conflict of Interest Statement

The authors declare that the research was conducted in the absence of any commercial or financial relationships that could be construed as a potential conflict of interest.

## Footnotes

1 http://www.ncbi.nlm.nih.gov/

2 http://genome.jgi.doe.gov/Wolco1/Wolco1.home.html

3 http://blast.ncbi.nlm.nih.gov/Blast.cgi?PROGRAM=blastx&PAGE_TYPE=BlastSearch&BLAST_SPEC=OGP__4932__9518&LINK_LOC=blasttab&LAST_PAGE=blastp

4 http://genome.jgi.doe.gov/Sclsc1/Sclsc1.home.html

## Supplementary Material

Table S1 **Protein kinase genes in *Wolfiporia cocos***.
